# Endocrine Disorders and Metabolic Dysfunction-Associated Steatotic Liver Disease: A Narrative Review

**DOI:** 10.3390/biomedicines13102500

**Published:** 2025-10-14

**Authors:** Joanna Betlejewska, Joanna Hubska, Zuzanna Roszkowska, Aleksandra Maciejczyk, Dominika Bachurska, Jan Domański, Maciej Miarka, Joanna Raszeja-Wyszomirska, Małgorzata Bobrowicz, Urszula Ambroziak

**Affiliations:** 1 Department of Internal Medicine and Endocrinology, Medical University of Warsaw, Banacha 1a, 02-097 Warsaw, Poland; joanna.betlejewska@wum.edu.pl (J.B.); joanna.hubska@wum.edu.pl (J.H.); dominika.bachurska@gmail.com (D.B.); jandomanski2001@gmail.com (J.D.); 2 Doctoral School of the Medical University of Warsaw, Medical University of Warsaw, 61 Zwirki i Wigury Street, 02-091 Warsaw, Poland; aleksandra.maciejczyk@wum.edu.pl; 3 Student Scientific Club “Endocrinus” Affiliated to the Department of Internal Medicine and Endocrinology, Medical University of Warsaw, Banacha 1a, 02-097 Warsaw, Poland; s082681@student.wum.edu.pl; 4 Department of Hepatology, Transplantology and Internal Medicine, Medical University of Warsaw, Banacha 1a, 02-097 Warsaw, Poland; maciej.miarka@wum.edu.pl (M.M.); joanna.wyszomirska@wum.edu.pl (J.R.-W.)

**Keywords:** liver disease, steatosis, hormones, endocrinology, metabolism, obesity

## Abstract

Metabolic dysfunction-associated steatotic liver disease (MASLD) is the hepatic manifestation of systemic metabolic dysregulation, strongly linked to type 2 diabetes, cardiovascular diseases, liver-related complications, and different types of malignancies. Although MASLD is associated with obesity and insulin resistance, it is increasingly recognized that the liver engages in complex crosstalk with several endocrine pathways, including thyroid function, sex and steroid hormone regulation, and growth hormone signaling. The pathophysiology of MASLD is multifactorial and complex, as reflected by its clinical range—from simple steatosis to cirrhosis. MASLD now affects about one-quarter of the global population, with its prevalence rising due to sedentary lifestyles, chronic caloric excess, and endocrine disturbances contributing to disease progression. Given the escalating prevalence of MASLD and its frequent concurrence with various endocrinopathies, understanding this relationship is critical for refining diagnostic accuracy and optimizing therapeutic strategies. This review aims to synthesize contemporary insights into the complex interactions between MASLD and selected endocrine disorders, elucidate underlying pathophysiological mechanisms, and underscore novel treatment paradigms. As MASLD remains a significant clinical challenge worldwide, its intersection with endocrine dysfunctions represents a vital and promising domain for future research and clinical management.

## 1. Introduction

Metabolic dysfunction–associated steatotic liver disease (MASLD) constitutes the hepatic manifestation of a multisystem disorder driven primarily by metabolic dysregulation. It is characterized by hepatic steatosis and is strongly associated with cardiometabolic and liver-related complications, including type 2 diabetes (T2DM), cardiovascular diseases, and several malignancies linked to lifestyle factors [1,2].

The historical concept of this condition dates back to 1980, when Ludwig et al. first described the histological features of nonalcoholic steatohepatitis (NASH), most often observed in patients with obesity or T2DM [3]. NASH was recognized as the progressive form of nonalcoholic fatty liver disease (NAFLD), which spans a spectrum from simple steatosis to steatohepatitis with fibrosis, cirrhosis, and hepatocellular carcinoma [1]. For decades, NAFLD was diagnosed by demonstrating ≥ 5% steatotic hepatocytes in the absence of significant alcohol intake or other chronic liver disease, and subclassified into nonalcoholic fatty liver (NAFL) and NASH [4].

Over time, this exclusion-based definition was increasingly criticized as insufficient since metabolic dysfunction rather than alcohol intake is central to pathogenesis. In 2020, NAFLD was renamed metabolic dysfunction–associated fatty liver disease (MAFLD), with diagnostic criteria requiring the presence of metabolic abnormalities [5]. Finally, in 2023, the nomenclature was further revised to MASLD and MASH, terms that better reflect the underlying pathophysiology and highlight the importance of metabolic risk factors [1,2,3].

The MASLD nomenclature emphasizes the centrality of metabolic dysfunction and no longer relies on excluding other etiologies, including alcohol consumption [1,2,3]. According to current diagnostic criteria, MASLD is established when hepatic steatosis is present in conjunction with at least one of the five following cardiometabolic criteria: overweight/obesity or increased waist circumference, prediabetes/T2DM, hypertension, hypertriglyceridemia, or low HDL-cholesterol [2,6]. Obesity and insulin resistance (IR), both of which are driven by chronic positive energy balance, are central to MASLD pathogenesis [7]. When adipose tissue (AT) storage capacity is exceeded, ectopic lipid accumulation ensues, including in the liver.

MASLD affects approximately 25% of the global population [1,5]. The global burden of MASLD continues to rise, largely in response to increasingly sedentary lifestyles, reduced physical activity, and chronic caloric excess from energy-dense, nutritionally imbalanced diets [8]. Notably, poor metabolic health is now frequently observed even among individuals with normal body weight, particularly in high-income settings [2,6].

Socioeconomic and healthcare inequalities strongly affect MASLD outcomes [2,5]. In countries with a lower socio-demographic index, a measure combining income per capita, average educational attainment, and fertility rates, limited access to primary care, low screening rates, and delayed diagnoses increase mortality and disability. Food insecurity, poverty and low health literacy promote reliance on calorie-rich, nutrient-poor diets, raising the risk of obesity and T2DM [6,8]. Countries with a higher socio-demographic index benefit from better healthcare access and public health programs, but rising MASLD rates show that medical care alone is insufficient; lifestyle and dietary interventions are crucial [4].

In addition to well-recognized metabolic risk factors, a growing body of evidence implicates endocrine dysregulation—such as deficiencies in growth hormone (GH), sex hormones, and thyroid hormones as well as hypercortisolemia—in the development and progression of MASLD [9,10]. Although distinguishing between direct causal effects and those resulting from increased adipocyte mass can be challenging, there is considerable evidence that endocrine disturbances influence the severity of MASLD both directly and indirectly [7].

In this review, we explore the interplay between MASLD and selected endocrine disorders, with a focus on the GH axis, thyroid dysfunction, adrenal abnormalities, and sex hormones. While substantial literature supports associations between MASLD and various endocrinopathies (reviewed in [2,9,10,11]) the current evidence base is insufficient to issue formal recommendations for routine endocrine screening in all patients with MASLD. Conversely, MASLD is often underrecognized by both primary care physicians and endocrinologists. Similarly, hepatologists frequently receive limited training in endocrine pathophysiology, which may hinder comprehensive assessment, particularly in patients with persistent or atypical disease courses [5,10]. Few publications have integrated the recent nomenclature shift to MASLD and its clinical implications, or provided a comprehensive synthesis across diverse endocrine disorders, including polycystic ovary syndrome (PCOS), congenital adrenal hyperplasia (CAH), hypogonadism, thyroid dysfunction, growth hormone deficiency (GHD), and cortisol excess. Importantly, the literature still lacks clear identification of high-risk endocrine subgroups, uniform screening recommendations, and robust evidence on the impact of endocrine interventions on hepatic outcomes.

This review aims to provide guidance on when to consider MASLD in the diagnostic workup of newly identified endocrine disorders, and conversely, when to evaluate for underlying endocrinopathies in patients with MASLD—especially those not responding to standard interventions. Timely identification of MASLD is essential to prevent progression to advanced liver disease, including fibrosis and cirrhosis.

## 2. Methodology

We performed a structured literature search of PubMed, Scopus and Web of Science covering years 2000–2025, with an emphasis on publications from the last 5 years. The following keywords were used: MASLD, MAFLD, NAFLD, nonalcoholic fatty liver disease, steatotic liver disease, metabolic dysfunction-associated steatotic liver disease, metabolic liver disease, endocrine disorders, thyroid dysfunction, hypothyroidism, hyperthyroidism, polycystic ovary syndrome, hyperandrogenism, congenital adrenal hyperplasia, hypercortisolism, Cushing syndrome, mild autonomous cortisol secretion, hypogonadism, testosterone deficiency, menopause, Turner syndrome, Klinefelter syndrome, insulin resistance, glucose metabolism, and lipid metabolism. Eligible studies included original research articles, systematic and narrative reviews, meta-analyses, and clinical guidelines relevant to the association between MASLD and endocrine disorders. Titles and abstracts were first screened for relevance, followed by full-text evaluation of potentially eligible studies. Articles not written in English, case reports, conference abstracts, and studies unrelated to MASLD or endocrine comorbidities were excluded.

## 3. Pathophysiology and Long-Term Consequence of MASLD

The pathophysiology of MASLD is multifactorial and complex, as reflected by its clinical range—from simple steatosis to cirrhosis, representing the terminal stage of liver disease. Various metabolic disturbances contribute to hepatic alterations. Earlier, its pathogenesis was explained by the “two-hit” hypothesis: the first step involving intra-hepatic accumulation of fatty acids, and the second encompassing additional insults such as oxidative stress or mitochondrial injury. This model, however, is now regarded as overly simplistic. It has been replaced by the “multiple-parallel hits” concept, which better reflects the synergistic actions of various factors, particularly in genetically pre-disposed individuals [12,13,14]. According to this model, genetic susceptibility combined with environmental influences and dietary patterns promotes obesity, IR, and alterations in the gut microbiome [12].

**Figure 1:**
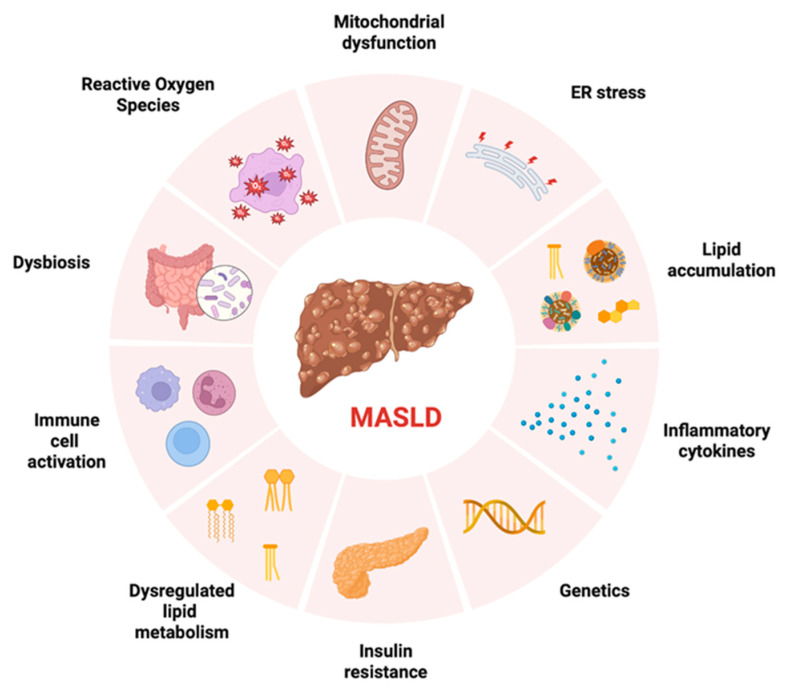
Schematic representation of the multifactorial pathogenesis of metabolic dysfunction–associated steatotic liver disease (MASLD). Contributing mechanisms include mitochondrial dysfunction, endoplasmic reticulum (ER) stress, lipid accumulation, pro-inflammatory cytokine release, genetic predisposition, insulin resistance, dysregulated lipid metabolism, immune cell activation, alterations in gut microbiota composition (dysbiosis), and increased production of reactive oxygen species. These interconnected processes promote hepatic steatosis, inflammation, and fibrogenesis, driving disease progression. Created with BioRender.

A key initiating element of MASLD is the accumulation of lipid droplets in hepatocytes due to elevated free fatty acids (FFAs), from both diet and increased lipolysis in AT as a consequence of energetic surplus and metabolic disturbances leading to IR [14,15]. Excess hepatic fatty acids induce mitochondrial impairment, endoplasmic reticulum stress, oxidative damage, and reactive oxygen species generation [16]. In parallel, gut microbiome dysregulation increases intestinal fatty acid production and permeability [17]. At the same time, dysregulation of the gut microbiome has emerged as an important driver of disease. Altered microbial composition increases intestinal permeability and facilitates the translocation of bacterial endotoxins, which activate inflammatory mediators such as TNF-α and IL-6. Specific features of dysbiosis, including an elevated Firmicutes/Bacteroidetes ratio and overgrowth of Proteobacteria and Enterobacteriaceae, further promote metabolic endotoxemia and chronic low-grade inflammation [17,18,19,20]. Another contributing factor to MASLD is the upregulation of hepatic de novo lipogenesis, stimulated by excessive carbohydrate consumption and elevated insulin levels. In addition, IR impairs AT function, leading to the secretion of pro-inflammatory cytokines [21,22]. Together, these processes lead to hepatic fat overload, promoting metabolic stress, mitochondrial dysfunction, and increased susceptibility to lipotoxicity and inflammation [23]. All the aforementioned factors acting simultaneously support the multiple-hit hypothesis, which has been proposed to explain the pathogenesis of this disease [23] (Figure 1).

**Figure 2:**
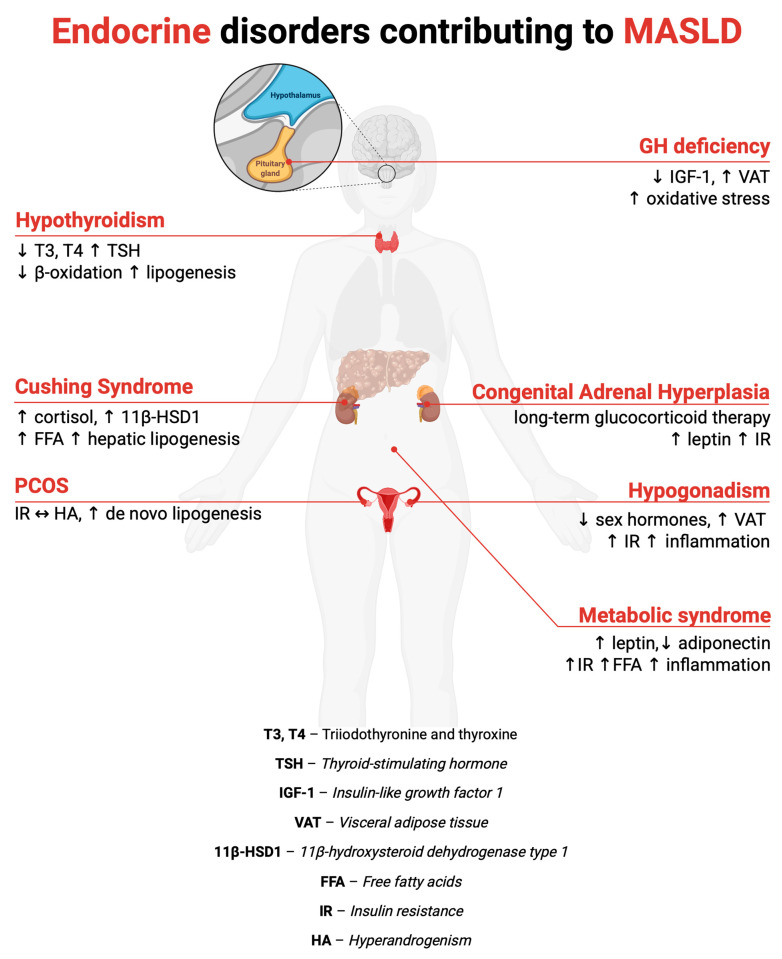
Endocrine disorders contributing to the development of metabolic dysfunction–associated fatty liver disease (MASLD) and their proposed pathogenic mechanisms. Created with BioRender. The arrows indicate the direction of change in metabolic/hormonal parameters (increase ↑ or reduction ↓). The horizontal arrow denotes the proposed pathophysiological link.

Beyond metabolic dysregulation, hormonal disturbances contribute to the development and progression of MASLD [6]. The liver functions as a central target and modulator of multiple hormonal axes, and disturbances in their regulation may directly impair hepatic lipid handling, insulin signaling, and inflammatory homeostasis [24]. Various endocrinopathies—both primary disorders and those developing secondary to other diseases—can contribute to the development and progression of MASLD. These include, among others, PCOS [25], hypercortisolism [26], CAH [27], hypogonadism [28], hypothyroidism [29], GHD [30] (Figure 2). In each of these conditions, distinct yet partly overlapping mechanisms are activated, which, through their effects on lipid metabolism, insulin sensitivity, and the regulation of inflammatory and fibrotic processes, promote hepatic steatosis and liver fibrogenesis. These mechanisms encompass alterations in AT distribution and function, changes in sex steroid and glucocorticoid (GC) bioavailability, dysregulation of anabolic and catabolic hormonal signaling, and modulation of pro-inflammatory and pro-fibrotic pathways.

Although traditionally seen primarily as a liver disease, MASLD may have long-term systemic consequences as presented by the recent research (excellently reviewed in [31,32]). The individuals affected with the disease show additional complications concerning in particular the cardiovascular, muscular, and renal systems. It has been postulated that the pro-inflammatory mediators produced in the liver disseminate through the bloodstream to distant organs resulting in the exacerbation of the already developing pathological processes in the heart, skeletal muscle, and kidneys [12]. In line, MASLD has been linked to systemic consequences such as cardiovascular dysfunction—coronary artery disease, heart failure, arrhythmias, sarcopenia, and impairment of kidney function. These systemic effects not only worsen the overall disease burden but also significantly affect patient outcomes by increasing morbidity and mortality. The progression of MASLD to MASH is highly variable between the studies; however it is accepted that risk factors include alcohol consumption and the elements of metabolic syndrome. Importantly, MASLD is a risk factor for hepatocellular carcinoma (HCC), although HCC is present in a small percentage of these patients. With the anticipated increase in the prevalence of MASLD in the future, it has been suggested that the disease will become the major indication for liver transplantation. Considering that, it is important to actively monitor the patients for this entity.

## 4. MASLD in Selected Endocrine Disorders

### 4.1. Obesity and the Metabolic Syndrome

Obesity is now increasingly recognized not only as a metabolic disorder but also as a complex, endocrine-related disease with systemic consequences. According to the World Health Organization, over 650 million adults globally are affected by obesity, 1 in 8 people worldwide [33,34,35]. Consequently, obesity emerges as one of the most important modifiable risk factors for the development of MASLD, particularly when accompanied by features of the metabolic syndrome [36,37]. It impairs hormonal regulation through the endocrine activity of AT, which secretes adipokines and inflammatory cytokines that promote IR, dyslipidemia, and systemic inflammation [38]. Beyond its role as the primary energy reservoir, AT produces hundreds of secretory factors that regulate metabolism, food intake, and immune cell function [39,40]. AT comprises not only adipocytes but also a complex network of stromal vascular fraction cells [41], resident immune cells [42], extracellular matrix [43] and nerve fibers [44,45]. In a coordinated response, these cellular components secrete bioactive molecules including adipokines, pro-inflammatory cytokines, lipokines, and extracellular vesicles that mediate endocrine signaling pathways across multiple organ systems [45,46]. White adipose tissue (WAT), the main depot, produces hormones and signaling molecules such as leptin, adiponectin, visfatin, resistin, apelin, and chemerin, as well as inflammatory mediators such as tumor necrosis factor-α (TNF-α), monocyte chemoattractant protein-1 (MCP-1), and plasminogen activator inhibitor-1 (PAI-1) [47,48,49]. These agents regulate insulin sensitivity and immune activation at both local and systemic levels [50,51]. By contrast, brown adipose tissue (BAT), although less abundant in adults, contributes to metabolic regulation via thermogenic activity [52] and the secretion of batokines including fibroblast growth factor 21, neuregulin-4, and IL-6, thereby linking adipose-derived endocrine signaling to total energy expenditure [53,54].

In obesity, particularly with excess visceral fat, the regulatory function of AT becomes pathologically altered [55]. Levels of adiponectin, an adipokine with anti-inflammatory and hepatoprotective effects, are reduced and inversely correlate with hepatic steatosis and fibrosis [56]. Concurrently, leptin and other pro-inflammatory adipokines rise sharply [57]. Chronic hyperleptinemia promotes hepatic stellate cell activation and fibrogenesis [58,59], while elevated chemerin and retinol-binding protein 4 (RBP4) levels have been shown to be associated with increased liver fat content, IR and adverse metabolic profiles [60,61]. In parallel, as visceral fat increases, more macrophages enter the AT, inducing a phenotypic switch from anti-inflammatory M2 to pro-inflammatory M1 macrophages [62,63]. As a result, the secretion of TNF-α, IL-6, and MCP-1 is amplified [62], which collectively impairs insulin receptor signaling through serine phosphorylation of insulin receptor substrate 1 (IRS-1) and insulin receptor substrate 2 (IRS-2) [64,65,66]. Consequently, systemic IR progresses, promoting lipolysis and increasing the portal delivery of FFAs to the liver [67,68]. Once in the liver, FFAs and cytokines activate transcription factors such as sterol regulatory element-binding protein-1c (SREBP-1c) and carbohydrate response element-binding protein (ChREBP), promoting de novo lipogenesis and triglyceride synthesis, thus leading to the progression of MASLD [51,69].

### 4.2. Growth Hormone Axis Dysfunction

The growth hormone (GH)/insulin-like growth factor-1 (IGF-1) axis is central to systemic metabolic regulation, significantly impacting various organs, including the liver [70,71,72,73]. In response to pituitary GH release, hepatocytes synthesize approximately 80% of circulating IGF-1 [72]. Furthermore, GH directly promotes lipolysis in WAT, elevating free fatty acid (FFA) flux to the liver and modulating insulin sensitivity [72,74]. In the setting of GHD, patients develop characteristic features of the metabolic syndrome: marked visceral adiposity, dyslipidemia, reduced muscle mass and strength, which strongly predispose patients to the development of MASLD [70,74,75].

Clinical data support this association. In the Hypopituitary Control and Complications Study, which included a large cohort of 2531 patients with severe GHD, metabolic syndrome was identified in 42.3% of participants [30]. More recently, a cross-sectional study involving 76 individuals with childhood-onset hypopituitarism demonstrated a higher prevalence of hepatic steatosis and fibrosis that correlated with HOMA-IR and waist circumference [76]. Several studies found that hepatocyte-specific GHR deletion leads to impaired hepatic regeneration and rapidly induces steatosis, even in the absence of systemic IR [71,77,78,79]. Liver-specific deletion of JAK2 or STAT5 in murine models also resulted in massive steatosis, accumulation of reactive oxygen species, and accelerated hepatocellular tumorigenesis, thereby highlighting the protective role of the GHR–JAK2–STAT5 axis [80]. In parallel, a proteometabolomic study in pituitary GHD found that overexpression of CYP2E1/CYP4A and depletion of NADPH-generating pathways contribute to oxidative stress and lipid peroxidation, potentially accelerating the progression of MASLD [81].

In contrast to GHD, acromegaly is a disorder characterized by chronic GH excess [82], associated with alterations in lipid and glucose metabolism [83,84]. Although GH promotes lipolysis and induces systemic IR [85], its role in hepatic lipid homeostasis appears unclear (79). Individuals with active acromegaly typically exhibit reduced visceral adipose tissue and greater lean body mass, features generally associated with a lower metabolic risk profile [86]. While the exact mechanisms remain undefined, elevated hepatic ATP synthesis may play a contributory role [87]. Several studies reported that biochemical control of acromegaly is associated with a redistribution of fat mass, notably an increase in hepatic lipid content, underlying a protective role of GH against steatosis [88,89]. Sustained increases in visceral and subcutaneous adipose tissue as well as intrahepatic lipids have been observed following surgical intervention, in parallel with the improvement in IR [89]. Moreover, a lower prevalence of MASLD has been reported in patients with active acromegaly compared to those with controlled disease using magnetic resonance imaging-proton density fat fraction, underscoring the importance of treatment strategies that maintain disease control without inducing GHD [84]. Larger cohort studies are needed to identify the key determinants of MASLD in patients with acromegaly [84,88].

### 4.3. Polycystic Ovary Syndrome

PCOS is one of the most common endocrine disorders in women of reproductive age, affecting approximately 8–13% of this population [90,91]. Notably, up to 70% of cases remain undiagnosed [91]. There is a strong association between PCOS and MASLD, two metabolic disorders that often coexist and share overlapping pathophysiological mechanisms [92,93,94]. The prevalence of MASLD among women with PCOS ranges from 23.8% to 43% across studies [95,96,97]. Multiple mechanisms contribute to this comorbidity, including obesity, HA, IR, chronic low-grade inflammation, and genetic factors [98,99,100]. Higher body mass index (BMI) in women with PCOS is strongly associated with increased MASLD, with obese patients showing the highest rates [97]. Another study confirmed that higher BMI strongly increases MASLD risk in women with PCOS, but the risk is also elevated in lean PCOS women, indicating an effect independent of obesity [96]. Despite ongoing debates regarding the diagnostic criteria for PCOS [90,101], excess androgen production remains a defining hallmark of the condition [102,103,104]. Several studies demonstrated that HA is an independent risk factor for MASLD in this population of patients [105,106]. Women with PCOS and HA exhibited more severe hepatic steatosis than their normoandrogenic counterparts, independent of obesity and IR [107]. IR is another significant feature of PCOS, affecting 50–70% of women with central obesity and up to 30% of lean PCOS patients [108,109]. IR and HA form a vicious cycle that both drives and results from metabolic and hepatic dysfunction [110,111]. In response to IR, compensatory hyperinsulinemia develops and acts alongside luteinizing hormone (LH) at the ovarian level, functioning as a co-gonadotropin [112,113] leading to increased androgen production and secretion [114]. In parallel, insulin acts beyond the ovary; it increases LH pulsatility, promotes adrenal steroidogenesis via P450c17α, and suppresses hepatic production of sex hormone-binding globulin (SHBG), thereby elevating the concentration of free androgens [114,115]. Low SHBG levels not only exacerbate the phenotypic features of PCOS but are also implicated in the development of MASLD [95,116,117]. Specifically, in a MASLD mouse model the overexpression of SHBG suppressed hepatic lipogenesis and reduced liver fat accumulation [118]. Low SHBG levels have been linked to higher liver fat, consistent with data showing SHBG overexpression reduces steatosis [119,120]. Experiments in HepG2 cells (human hepatocyte model) showed that SHBG activates the ERK1/2–MAPK pathway, leading to reduced peroxisome proliferator–activated receptor-γ expression and subsequent downregulation of lipogenic enzymes, including acetyl-CoA carboxylase [121]. Additionally, in an in vitro study on macrophages and adipocytes, physiological SHBG concentrations attenuated inflammation and lipid accumulation [122], suggesting that reduced SHBG may favor lipid storage in the liver. Chronic HA was demonstrated to promote IR and hepatic fat accumulation by impairing mitochondrial function, promoting hepatocyte apoptosis, and disrupting autophagy balance [123]. Moreover, androgens impair mitochondrial β-oxidation, enhance de novo lipogenesis, and trigger hepatic inflammation through upregulation of pro-inflammatory cytokines such as IL-6, TNF-α, MCP-1, and IL-1β [124].

### 4.4. Congenital Adrenal Hyperplasia

CAH is an autosomal recessive disorder, most commonly caused by 21-hydroxylase deficiency, leading to impaired adrenal steroidogenesis characterized by cortisol deficiency and androgen excess. Standard management involves supraphysiological doses of GCs to suppress adrenal androgen production; however, this approach is associated with an increased risk of obesity, hyperinsulinemia, and gestational diabetes [125]. The prevalence of obesity in both classic and non-classic forms of CAH has been reported to range between 30% and 40%. These patients are also prone to developing metabolic syndrome, with MASLD representing its hepatic manifestation. The underlying mechanisms likely include a predisposition to AT accumulation, dysregulation of adipokines, and elevated leptin levels [27]. Metabolic disturbances in CAH and PCOS exhibit notable similarities despite distinct etiologies. Both conditions are marked by hyperandrogenism, which contributes to IR, a central driver of adverse metabolic outcomes. IR is frequently observed in these patients, often independent of obesity, and increases the risk of T2DM, dyslipidemia, and cardiovascular disease [126,127]. Central adiposity is common in CAH and PCOS, and reduced levels of SHBG further exacerbate hyperandrogenism and IR, promoting metabolic complications such as MASLD [21,120,128]. In CAH, long-term GC therapy represents an additional risk factor, as it contributes to visceral fat accumulation and worsens IR [26,27,129]. In women with PCOS, an increased prevalence of MASLD has been consistently reported; however, studies directly assessing hepatic steatosis and fibrosis in this population remain limited.

However, given the shared endocrine and metabolic features between PCOS and CAH, it is reasonable to hypothesize that individuals with CAH may also be at increased risk of developing MASLD. A Swedish study examined liver enzyme profiles in 61 women with CAH compared with healthy controls. Serum alanine aminotransferase (ALT) and gamma-glutamyl transferase (GGT) levels were significantly higher in the CAH group, while alkaline phosphatase (ALP) levels approached statistical significance (*p* = 0.052). Aspartate aminotransferase (AST) did not differ significantly. Notably, enzyme elevations were more pronounced in the subgroup of patients aged ≥30 years (AST, GGT, and ALP: *p* = 0.035, 0.007, and 0.045, respectively). Interestingly, even when restricting the analysis to non-obese individuals (defined as BMI < 30 kg/m^2^ and waist circumference ≤ 88 cm), liver function tests (LFTs) remained elevated in CAH patients compared to controls. These findings suggest that factors beyond central obesity—possibly including long-term GC exposure—may contribute to hepatic enzyme alterations in this population [130].

It should be emphasized that no imaging studies or liver biopsies were performed in this cohort, which limits the ability to confirm MASLD. This is clinically relevant, as liver enzymes may remain within normal limits despite histologically evident steatosis or fibrosis, leading to underdiagnosis when relying solely on biochemical markers [2,5]. Identification of high-risk groups is therefore essential to optimize screening strategies, which should include both imaging modalities and validated serum-based biomarkers.

### 4.5. Thyroid Dysfunction

Thyroid hormones are key regulators of hepatic lipid metabolism, influencing β-oxidation, lipogenesis, and cholesterol balance [11,131,132]. Hypothyroidism and hyperthyroidism can significantly affect liver physiology and contribute to the development of MASLD [133].

Hypothyroidism, both overt and subclinical, has been increasingly recognized as a factor contributing to intrahepatic lipid accumulation [134,135] and progression of MASLD [131,136]. Thyroid hormone deficiency reduces β-oxidation and hepatic lipase activity, enhances lipogenesis, and promotes oxidative stress and chronic inflammation [29,137]. Additionally, elevated thyroid-stimulating hormone (TSH) levels may directly stimulate hepatic lipogenesis via activation of SREBP-1c and suppression of AMP-activated protein kinase (AMPK) activity [137,138,139]. These mechanisms together create a metabolic environment favoring hepatic fat accumulation. Epidemiological studies provide substantial support for this association. A large 2018 meta-analysis, which included over 37,000 individuals from 13 observational studies, demonstrated that both overt and subclinical hypothyroidism were independently associated with MASLD (OR 1.52, 95% CI 1.24–1.87) [140]. A recent meta-analysis of 26 studies comprising 61,548 participants confirmed that hypothyroidism was significantly associated with a higher risk of MASLD and that patients with MASLD had higher TSH levels compared to controls [29]. The Rotterdam Study, a large population-based cohort of nearly 9500 individuals, further supported these findings by showing that both subclinical and overt hypothyroidism were significantly associated with MASLD as diagnosed by ultrasonography, independent of confounders such as age, BMI, diabetes, and lipid profile [141]. In a cohort of 232 euthyroid patients with T2DM, lower free thyroxine (FT4) levels were correlated with increased hepatic fat content on MR spectroscopy, although no direct association with liver histology was found [139]. In contrast, a large longitudinal study involving 18,500 South Korean individuals found no significant relationship between hypothyroidism and the risk of incident MASLD after adjusting for multiple cardiometabolic factors [142]. These discrepancies may be explained by differences in study design, diagnostic criteria for MASLD, and population characteristics, such as ethnicity, degree of obesity, or prevalence of metabolic comorbidities. Moreover, while some studies focus on cross-sectional associations, others assess longitudinal risk, which may account for divergent findings. It is also possible that thyroid dysfunction interacts with other metabolic pathways differently across populations, further contributing to heterogeneous results.

Hyperthyroidism appears to play a less prominent but potentially beneficial role in hepatic metabolism; however it needs to be emphasized it is not an aim in MASLD patients. Excess thyroid hormones increase lipolysis, elevating FFA levels delivered to the liver [131,133]. A case report described a MASLD patient whose liver enzymes improved during Graves’ disease and worsened after returning to euthyroidism, suggesting a possible protective effect of thyroid hormones [143]. In euthyroid individuals, subtle variations in thyroid hormone levels may also influence MASLD risk. There is a positive correlation between free triiodothyronine (FT3) and TSH levels and MASLD prevalence in euthyroid subjects [144]. A single study reported that MASLD patients presented with higher FT3 and lower FT4 concentrations, with no differences in TSH levels [145]. Some studies, especially in obese populations [146], support these findings, but others show no clear link, pointing to inconsistent results and the need for further research [147]. These conflicting observations likely reflect differences in study methodology, hormonal assay sensitivity, and the metabolic profile of studied cohorts. Variations in obesity rates, IR, and genetic background may influence the interplay between thyroid hormones and hepatic lipid metabolism, leading to inconsistent outcomes. Consequently, while hyperthyroidism might exert transient protective effects, the role of subtle thyroid hormone variations in euthyroid individuals remains uncertain and requires further clarification.

This reflects the heterogeneity among observational studies, likely due to differences in population characteristics and study design.

### 4.6. Hypogonadism

Hypogonadism is considered a risk factor for MAFLD [148]. Reduced secretion of sex hormones—estrogens in women and testosterone in men—can disrupt lipid and glucose homeostasis, resulting in altered body composition [149]. Hormone levels may decline physiologically, as in menopause, or be pathologically low as in conditions such as Turner syndrome (TS) or Klinefelter syndrome.

In men, conditions such as hypogonadotropic or primary hypogonadism—including Klinefelter syndrome or acquired testosterone deficiency—are associated with increased hepatic fat accumulation, unfavorable lipid profiles, and elevated inflammatory markers. On the contrary, adipocytes produce leptin and pro-inflammatory factors that inhibit the production of testosterone. Indeed, epidemiologic studies mention that lower testosterone levels are correlated with the prevalence of MASLD [150,151].

Estrogens increase insulin sensitivity, promote fatty acid oxidation, and suppress hepatic lipogenesis via estrogen receptor-α pathways. Liver abnormalities among TS patients such as the elevation of aminotransferases, GGT and ALP [152], are detected mostly throughout systematic blood testing at asymptomatic stage. Studies have reported various histological changes among this group of varying from minimal changes to steatosis, steatohepatitis or cirrhosis [28]. This low-grade chronic inflammation contributes to hepatocellular injury, fibrogenesis, and the progression from simple steatosis to steatohepatitis and fibrosis [153,154]. Additionally, estrogen deficiency—which is a hallmark of TS due to gonadal dysgenesis [155]—can contribute to metabolic dysfunction. Estrogens have been shown to have protective effects on hepatic fat accumulation and insulin sensitivity [156]; their absence may therefore accelerate the development and progression of MASLD in groups with declined estrogens. Also, in postmenopausal women and plausibly in those with primary ovarian insufficiency, the estrogen deficiency contributes to the pathogenesis of MASLD [157,158,159]. While hormone replacement therapy has been proposed as a potential intervention, inconsistent study outcomes underscore the urgent need for rigorously designed clinical trials.

### 4.7. Hypercortisolism

GCs, play a pivotal role in lipid metabolism, fat distribution, and the pathogenesis of MASLD [133,160]. Notably, the prevalence of MASLD in hypercortisolemic patients has not been sufficiently documented (reviewed in [160]), despite the clear image of the impact of hypercortisolemia on the metabolic syndrome.

GCs may contribute to MASLD development primarily through their lipolytic action on AT, increasing the availability of FFAs for hepatic uptake. While GCs alone suppress lipogenesis, their combined effect with insulin is synergistic, promoting lipid accumulation in the liver [133,161]. Excessive GC levels activate GC receptors in adipocytes, triggering molecular pathways that promote hepatic steatosis and IR. This receptor activation enhances lipolysis and hinders the proper expansion of AT, leading to ectopic fat deposition and broader metabolic dysfunction.

Conversely, the immunosuppressive properties of GCs may attenuate intrahepatic low-grade chronic inflammation, largely mediated by interleukin-6 (IL-6), which could partly explain the relatively low prevalence of MASLD reported in overt Cushing syndrome in some studies [162]. In contrast, other studies suggest increased prevalence of MASLD not only in Cushing syndrome but also in mild autonomous cortisol secretion (MACS) and non-functioning adrenal adenomas [163]. Importantly, pharmacological targeting of the GC receptor has emerged as a potential therapeutic strategy for patients with hypercortisolemia and MASLD, as suggested by several case reports and preclinical studies [164,165,166].

## 5. Diagnostic Tools for the Assessment of Liver Function to Be Used in Endocrinological Patients

The evaluation of liver disease relies on both laboratory tests and non-invasive imaging methods to assess steatosis and fibrosis. Often, the finding of MASLD comes from a routine imaging examination – ultrasound, CT or MRI [167]. The degree of fibrosis can be further assessed by a transient elastography, the so-called a FibroScan, an ultrasound transducer mounted on a vibrating axis generates low-frequency vibrations that induce shear waves in the tissue. Pulse-echo ultrasound tracks the wave’s propagation, with its velocity reflecting tissue stiffness—the stiffer the tissue, the faster the wave [168]. The clinical interpretation of results should be performed by an experienced clinician and must be complemented by additional data, such as patient demographics, underlying disease etiology, and key laboratory findings to minimize the risk of misinterpretation. Although liver biopsy remains the gold standard for diagnosing MASLD, a meta-analysis has shown that the sensitivity and specificity of FibroScan in assessing both steatosis and fibrosis stages exceed 70% [169,170,171]. The American Association for the Study of Liver Disease recommends the use of liver elastography with the FibroScan technique as a screening tool for the detection of hepatic steatosis in individuals with risk factors for MASLD, including overweight, obesity, elevated serum lipid levels, T2DM and arterial hypertension [172].

In everyday practice, as the general practitioner, the simple non-invasive scoring systems such as FIB-4 can be used as initial screening tools. The FIB-4 index is a well-established, non-invasive index of the severity of liver fibrosis that is calculated using age, platelet count, and AST and ALT [173]. Additional non-invasive methods include the AST to platelet ratio index (APRI), the AST/ALT ratio, and the NAFLD Fibrosis Score (NFS). The NFS is based on a regression model incorporating six variables: age, BMI, impaired fasting glucose or diabetes, AST/ALT ratio, platelet count, and serum albumin level [174]. The European Association for the Study of the Liver (EASL) recommends the FIB-4 index as the preferred first-line screening tool for liver fibrosis, while the NFS is useful for identifying patients who require referral for a specialist [175].

The non-invasive and imaging scoring systems are summarized in the table below (Table 1).

## 6. Treatment of MASLD

While the correction of the underlying hormonal imbalance is essential, it is rarely sufficient to fully reverse liver pathology [133]. For instance, although restoring euthyroidism in hypothyroid patients can normalize lipid levels and improve hepatic parameters, significant liver improvement is typically observed only when accompanied by broader metabolic control [176]. Alongside therapies aimed at correcting hormonal disturbances, the non-pharmacological interventions such as ≥5–10% weight loss [177], increased physical activity [1], and adherence to a Mediterranean diet [178] are essential components of MASLD management. Recently, some reports on the use of very low-calorie ketogenic diets suggest potential benefit in reversing liver steatosis, but definitive proofs are still lacking [179,180]. In all MASLD patients refraining from heavy alcohol consumption, alcohol abstinence should be suggested. Moreover, pharmacologic therapies including glucagon-like peptide-1 (GLP-1) receptor agonists and peroxisome proliferator-activated receptor (PPAR) agonists are being actively investigated for their benefits on liver health. GLP-1 receptor agonists, such as semaglutide, improve insulin sensitivity, support weight loss, and reduce liver fat. A phase 2 trial showed that semaglutide led to resolution of MASLD in many patients, although its effect on fibrosis was limited [181]. Tirzepatide, a dual glucose-dependent insulinotropic polypeptide (GIP)/GLP-1 receptor agonist, has also demonstrated superior efficacy in MASLD resolution without fibrosis worsening, with more than half of patients achieving fibrosis improvement [182]. In preclinical MASLD models, tirzepatide reduced hepatic triglyceride and cholesterol content, downregulated lipid uptake proteins CD36 and odorant binding protein 2A, restored adipose triglyceride lipase (ATGL) expression, and improved histology without major off-target metabolic effects [183]. Clinical reports further indicate histological improvement even in advanced fibrosis refractory to other GLP-1 receptor agonists, alongside reductions in liver enzymes and visceral fat despite minimal weight loss [184]. PPAR agonists, which regulate lipid metabolism, inflammation, and fibrosis, have also shown promising results. Both PPARγ agonists and dual PPARα/δ agonists have improved liver histology in clinical trials [185].

While lifestyle modification remains the cornerstone of MASLD management in PCOS [186,187], pharmacologic strategies are increasingly being explored [188]. Among these, metformin, a long-established first-line therapy for T2DM, has shown promise for hepatic protection in PCOS [189]. Metformin exerts beneficial effects on dyslipidemia and IR, the primary drivers of MASLD in PCOS [190,191]. Metformin has been reported to effectively reduce hepatic lipid accumulation and attenuated hepatic injury in PCOS models [192]. Through mitochondrial mediated by the activation of the Ethe1/Keap1/Nrf2/PINK1/Parkin pathway, metformin appears to restore mitochondrial integrity and enhance liver recovery in murine PCOS models.

Furthermore, moderate dosing of metformin (50 mg/kg/day for two months) proved to be more effective than both lower (10 mg/kg/day) and higher (250 mg/kg/day) doses in reducing hepatic triglyceride accumulation and preserving mitochondrial function. This intermediate dosing achieved a greater reduction in hepatic triglyceride accumulation and better preservation of mitochondrial integrity [192]. Beyond these benefits, it has been suggested that metformin may also reduce the risk of progression to cirrhosis and decrease the incidence of hepatocellular and biliary cancers in preclinical studies [193]. These findings suggest a broader therapeutic role for metformin in MASLD among women with PCOS, although further research is needed to clarify its long-term efficacy in this population.

Novel endocrine-targeted agents also show promise. The thyroid hormone receptor (THR) β primarily expressed in the liver, has become an attractive target for steatosis reduction. Rodent studies demonstrate that selective THRβ agonists significantly decrease hepatic lipid accumulation [194], while mice lacking THRα are protected from diet-induced hepatic steatosis and IR [195]. These findings suggest potential therapeutic benefit from receptor-specific modulation of thyroid signaling in the liver.

Although direct adipokine targeting in MASLD is still limited today, evidence supports their modulation through lifestyle and pharmacologic strategies [58]. Sustained weight loss via bariatric surgery or dietary interventions reduces circulating leptin [196,197,198], resistin, RBP4, visfatin, and chemerin, while increasing adiponectin [199]. In a cohort of 294 patients with abdominal obesity/dyslipidaemia a polyphenol-rich Mediterranean diet decreased intrahepatic fat alongside leptin and chemerin levels [200]. Also, exercise-induced increases in adiponectin were associated with reductions in liver fat, as assessed by magnetic resonance imaging [201].

An emerging and promising direction in the treatment of MASLD involves modulation of the gut microbiota. Dysbiosis has been strongly linked to the pathogenesis of MASLD through increased intestinal permeability, bacterial translocation, and endotoxin-driven hepatic inflammation [201]. Microbial products such as lipopolysaccharides (LPS) activate hepatic Toll-like receptor 4 (TLR-4) signaling and promote Kupffer cell and hepatic stellate cell activation, thereby driving inflammation and fibrosis [202,203,204]. Patients with MASLD often exhibit small intestinal bacterial overgrowth (SIBO) and altered gut microbial profiles; these profiles include a reduced abundance of *Bifidobacterium bifidum* and *Lactobacillus* spp. [205] and an expansion of taxa such as Escherichia coli, Streptococcus, Dorea, and Bilophila [206].

Beyond profiling, specific microbial strains and metabolites have shown therapeutic potential. Indole derivatives such as indole-3-propionic acid (IPA) and indole-3-acetic acid (IAA), products of tryptophan metabolism, are reduced in MASLD patients and play anti-inflammatory and hepatoprotective roles. Administration of Bifidobacterium bifidum, which enhances IAA production, prevented steatosis and inflammation in mouse models through mechanisms including reinforcement of the gut barrier, increased hepatic β-oxidation, suppression of NF-κB signaling, and activation of mucosal immune tolerance via aryl hydrocarbon receptor signaling [206]. These findings highlight the therapeutic promise of microbiome-targeted strategies as complementary approaches for MASLD management. All the described therapeutic approaches are summarized in Table 2.

## 7. Discussion and Conclusions

Although several publications have addressed these connections, current evidence remains insufficient to formulate definitive screening recommendations or to clearly define the high-risk patient subgroups that would benefit from targeted diagnostic strategies. MASLD represents a significant global health challenge that is frequently underdiagnosed. The number of diagnosed cases is expected to rise in the coming years due to lifestyle changes and the growing prevalence of metabolic disorders worldwide. As highlighted in this review, patients with endocrine disease frequently suffer also from MASLD or are at risk of developing it.

The strenghs of this literature review lie in its comprehensive and interdisciplinary approach and integrating recent advances in hepatology and endocrinology. It also incorporates recent MASLD nomenclature, as well as new insights on its clinical implications. This work, unlike previous publications, covers a broad spectrum of endocrine disorders, making this review valuable for practicing clinicians. Furthermore, it emphasizes up-to-date epidemiological data, diagnostic strategies, and therapeutic perspectives, providing clinicians with practical guidance for patient management. Finally, by identifying gaps in current knowledge, it outlines important directions for future research and clinical practice.

Endocrinologists play a pivotal role in the management of MASLD due to their expertise in hormonal and metabolic regulation. Their responsibilities include early detection, assessment of metabolic risk, and coordination of multidisciplinary care. In the era of widely available non-invasive diagnostic techniques, elastography—an excellent alternative to invasive liver biopsy—may become a standard component of liver assessment in endocrinology practice. Furthermore, markers of metabolic dysfunction, such as HOMA-IR, lipid profiles, and HbA1c, together with scoring systems including the fibrosis-4 index (FIB-4), should complement the diagnostic process and facilitate the identification of patients at risk for MASLD.

Effective management of MASLD requires a comprehensive, multidisciplinary approach that integrates lifestyle interventions—particularly dietary modifications and increased physical activity—with pharmacological treatments when indicated. Given the complex interplay between hepatic, metabolic, and cardiovascular systems, close collaboration between endocrinologists, hepatologists, cardiologists, and primary care physicians is essential for optimal patient outcomes. As previously noted, there remains a lack of clinical studies specifically evaluating the relationship between MASLD and distinct endocrine disorders as well as effective treatments for the affected patients.

## Figures and Tables

**Table 1:** Overview of commonly used histological, serologic, and imaging-based tools for liver fibrosis assessment in MASLD and other chronic liver diseases, summarizing fibrosis staging ranges, key advantages, and limitations.

Score	Fibrosis Staging	Pros	Cons
FIB-4 Index	<1.3 = low >2.67 = high risk	- Endorsed by EASL/AASLD as first-line in MASLD - Requires only age, AST, ALT, platelets - High negative predictive value for excluding advanced fibrosis	- Poor discrimination between intermediate stages (F2 vs. F3) - Lower specificity in elderly - Indeterminate results in ~30% of patients, requiring further testing
NAFLD Fibrosis Score (NFS)	<–1.455 = low >0.676 = high risk	- Incorporates metabolic variables (BMI, IFG/diabetes) - Good excluding advanced fibrosis	- Complex formula, less accessible clinical practice - Reduced accuracy in young or lean NAFLD patients
APRI	>1 = significant >2 = cirrhosis	- Easy calculation from routine labs - Endorsed by WHO for HCV-related fibrosis in low-resource settings	- Limited utility in early fibrosis stages - Outperformed by FIB-4 in most comparative studies
FibroTest/FibroSure	Score 0–1 (F0–F4 equivalent)	- Integrates multiple biomarkers (α2M, GGT, haptoglobin) - Validated in various chronic liver diseases	- Expensive - Proprietary algorithm limits transparency - Affected by hemolysis, inflammation, Gilbert syndrome
FibroScan (TE)	<7 = low >12–14 = high risk	- Widely validated - Point-of-care, non-invasive - Fast (5–10 min) - Strong correlation with biopsy for ≥F3 fibrosis - Useful for longitudinal follow-up	- Results affected by BMI >30 kg/m^2^, hepatic congestion, acute inflammation - Operator training required
MR Elastography (MRE)	Quantitative (kPa)	- Most accurate non-invasive method - Good discrimination across all fibrosis stages - Less affected by obesity or inflammation	- High cost - Requires MRI facility and contrast - Limited availability, especially in non-tertiary centers
LiverMultiScan (MRI-based)	cT1 (fibrosis/inflammation surrogate)	- Simultaneous assessment of fibrosis, inflammation, and steatosis - No need for contrast - Strong correlation with histology - Non-invasive, reproducible, and quantitative	- High cost - Requires advanced MRI hardware and dedicated software - Limited availability outside tertiary centers - Less extensively validated than MRE for fibrosis staging
ARFI (US-based elastography)	Qualitative stiffness estimates	- Better reproducibility than conventional ultrasound-based elastography techniques - Provides spatial mapping of liver stiffness - Integrated into modern ultrasound systems - Non-invasive and relatively fast	- Dependent on operator skill and liver window - Lack of universally validated cut-offs and external calibration
2D-SWE (Two-dimensional shear wave elastography)	Qantitative (kPa)	- Provides quantitative, objective measurements in kPa - Real-time 2D mapping of liver stiffness over a wider region of interest - Can be performed during a standard ultrasound exam without additional equipment - Non-invasive, reproducible, and relatively fast	- Dependent on operator skill and liver window - Limited external validation and universally accepted cut-offs - Accuracy decreases with obesity, ascites, or high inflammation

**Table 2:** Targeted Approaches in MASLD Management.

Therapeutic Target	Mechanism of Action	Current Evidence
Thyroid hormones and receptors	Restoration of euthyroidism improves lipid metabolism and reduces hepatic steatosis	Rodent studies and clinical observations; THR-β agonists under investigation [194,195]
Insulin resistance and weight reduction	Improves hepatic lipid oxidation, reduces steatosis, inflammation, and fibrosis	Well-established benefits in clinical trials; key part of MASLD management [1,177,179]
GLP-1 receptor agonists	Enhance insulin sensitivity, reduce body weight, improve liver enzyme levels, and induce MASLD resolution	Phase II trial with semaglutide showed MASH resolution without fibrosis improvement [185]
PPAR agonists	Regulate lipid metabolism, inflammation, and fibrosis via nuclear receptor activation	Phase II trials show improvement in liver histology; multiple agents in development [181]
Gut microbiota	Decrease endotoxemia, strengthen gut barrier, increase anti-inflammatory metabolites (e.g., IAA, IPA)	Animal studies support benefit; human trials ongoing; specific strains (e.g., *Bifidobacterium bifidum* and *Lactobacillus* spp.) show promise; overgrowth of Escherichia, Streptococcus, Dorea and Bilophila has been linked to MASLD progression [206]

## Data Availability

Not applicable.

## Abbreviations

The following abbreviations are used in this manuscript:

ALT	Alanine aminotransferase
ALP	Alkaline phosphatase
APRI	Aspartate aminotransferase-to-platelet ratio index
AST	Aspartate aminotransferase
AT	Adipose tissue
ATGL	Adipose triglyceride lipase
BAT	Brown adipose tissue
BMI	Body mass index
CAH	Congenital adrenal hyperplasia
ChREBP	Carbohydrate response element-binding protein
CI	Confidence interval
EASL	European Association for the Study of the Liver
ER	Endoplasmic reticulum
ERK1/2	Extracellular signal-regulated kinases 1/2
FFA	Free fatty acid
FFAs	Free fatty acids
FIB-4	Fibrosis-4 index
FT3	Free triiodothyronine
FT4	Free thyroxine
GGT	Gamma-glutamyl transferase
GH	Growth hormone
GHD	Growth hormone deficiency
GIP	Glucose-dependent insulinotropic polypeptide
GLP-1	Glucagon-like peptide-1
HbA1c	Glycated hemoglobin
HA	Hyperandrogenism
HCC	Hepatocellular carcinoma
HDL	High-density lipoprotein
HOMA-IR	Homeostasis model assessment of insulin resistance
IAA	Indole-3-acetic acid
IGF-1	Insulin-like growth factor 1
IL-1β	Interleukin 1 beta
IL-6	Interleukin 6
IPA	Indole-3-propionic acid
IR	Insulin resistance
IRS-1	Insulin receptor substrate 1
IRS-2	Insulin receptor substrate 2
JAK2	Janus kinase 2
KS	Klinefelter syndrome
LFTs	Liver function tests
LH	Luteinizing hormone
LPS	Lipopolysaccharide
MACS	Mild autonomous cortisol secretion
MAFLD	Metabolic dysfunction-associated fatty liver disease (*transient nomenclature*)
MAPK	Mitogen-activated protein kinase
MASLD	Metabolic dysfunction-associated steatotic liver disease
MASH	Metabolic dysfunction-associated steatohepatitis
MCP-1	Monocyte chemoattractant protein-1
MRE	Magnetic resonance elastography
MRI	Magnetic resonance imaging
NADPH	Nicotinamide adenine dinucleotide phosphate (reduced form)
NAFL	Non-alcoholic fatty liver
NAFLD	Non-alcoholic fatty liver disease
NASH	Non-alcoholic steatohepatitis
NFS	NAFLD fibrosis score
NF-κB	Nuclear factor kappa-light-chain-enhancer of activated B cells
OR	Odds ratio
PAI-1	Plasminogen activator inhibitor-1
PCOS	Polycystic ovary syndrome
PINK1	PTEN-induced putative kinase 1
PPAR	Peroxisome proliferator-activated receptor
RBP4	Retinol-binding protein 4
SIBO	Small intestinal bacterial overgrowth
SHBG	Sex hormone-binding globulin
SREBP-1c	Sterol regulatory element-binding protein-1c
STAT5	Signal transducer and activator of transcription 5
T2DM	Type 2 diabetes mellitus
TE	Transient elastography
THRβ	Thyroid hormone receptor beta
TNF-α	Tumor necrosis factor alpha
TS	Turner syndrome
TSH	Thyroid-stimulating hormone
WAT	White adipose tissue
